# The TREC/KREC Assay for the Diagnosis and Monitoring of Patients with DiGeorge Syndrome

**DOI:** 10.1371/journal.pone.0114514

**Published:** 2014-12-08

**Authors:** Eva Froňková, Adam Klocperk, Michael Svatoň, Michaela Nováková, Michaela Kotrová, Jana Kayserová, Tomáš Kalina, Petra Keslová, Felix Votava, Hana Vinohradská, Tomáš Freiberger, Ester Mejstříková, Jan Trka, Anna Šedivá

**Affiliations:** 1 CLIP, Department of Paediatric Haematology/Oncology, 2nd Medical School, Charles University Prague and University Hospital Motol, Prague, Czech Republic; 2 Department of Immunology, 2nd Medical School, Charles University Prague and University Hospital Motol, Prague, Czech Republic; 3 Department of Pediatrics, 3rd Medical School, Charles University Prague and University Hospital Kralovske Vinohrady, Prague, Czech Republic; 4 Department of Clinical Biochemistry, Children Hospital, Faculty of Medicine, Masaryk University Brno, Brno, Czech Republic; 5 Department of Clinical Immunology and Allergology, Medical Faculty, and Central European Institute of Technology, Masaryk University, Brno, Czech Republic; 6 Molecular Genetics Lab, Centre for Cardiovascular Surgery and Transplantation, Brno, Czech Republic; University of Thessaly, Faculty of Medicine, Greece

## Abstract

DiGeorge syndrome (DGS) presents with a wide spectrum of thymic pathologies. Nationwide neonatal screening programs of lymphocyte production using T-cell recombination excision circles (TREC) have repeatedly identified patients with DGS. We tested what proportion of DGS patients could be identified at birth by combined TREC and kappa-deleting element recombination circle (KREC) screening. Furthermore, we followed TREC/KREC levels in peripheral blood (PB) to monitor postnatal changes in lymphocyte production.

**Methods:**

TREC/KREC copies were assessed by quantitative PCR (qPCR) and were related to the albumin control gene in dry blood spots (DBSs) from control (n = 56), severe immunodeficiency syndrome (SCID, n = 10) and DGS (n = 13) newborns. PB was evaluated in DGS children (n = 32), in diagnostic samples from SCID babies (n = 5) and in 91 controls.

**Results:**

All but one DGS patient had TREC levels in the normal range at birth, albeit quantitative TREC values were significantly lower in the DGS cohort. One patient had slightly reduced KREC at birth. Postnatal DGS samples revealed reduced TREC numbers in 5 of 32 (16%) patients, whereas KREC copy numbers were similar to controls. Both TREC and KREC levels showed a more pronounced decrease with age in DGS patients than in controls (p<0.0001 for both in a linear model). DGS patients had higher percentages of NK cells at the expense of T cells (p<0.0001). The patients with reduced TREC levels had repeated infections in infancy and developed allergy and/or autoimmunity, but they were not strikingly different from other patients. In 12 DGS patients with paired DBS and blood samples, the TREC/KREC levels were mostly stable or increased and showed similar kinetics in respective patients.

**Conclusions:**

The combined TREC/KREC approach with correction via control gene identified 1 of 13 (8%) of DiGeorge syndrome patients at birth in our cohort. The majority of patients had TREC/KREC levels in the normal range.

## Introduction

DiGeorge syndrome is a complex disorder caused by an embryopathy; it presents with a number of clinical symptoms arising from the disturbed development of the 3^rd^ and 4^th^ pharyngeal arches, chief amongst which are congenital heart, great vessel and parathyroid gland defects as well as a typical malformation of the face and soft palate. The embryopathy is most commonly caused by a deletion on the 22^nd^ chromosome, called del22q11, but can be caused by other mutations as well [1]. The unifying sign of DiGeorge syndrome is a wide spectrum of pathologies of the thymus, ranging from complete athymia, found in complete DiGeorge syndrome patients, to various degrees of thymic dysplasia and disturbed migration of the thymus observed in partial DiGeorge syndrome patients [2].

The degree of thymic pathology correlates with various degrees of T-lymphocytic dysfunction [3]. The thymus is the location of the T-lymphocyte maturation and selection process. In children, it is a dominant place of T-lymphocyte development and a source of naïve T cells [4]. The number of naïve T-lymphocytes that went through the thymic maturation process can be assessed from peripheral blood using T cell receptor excision circle (TREC) detection by quantitative PCR [5]. In DiGeorge syndrome, the immunodeficiency is caused by dysplasia of the thymus, and it would therefore be logical to expect abnormal development of T-cells in the thymus and accordingly abnormal TREC levels. This assumption was partially proven to be correct with the introduction of SCID (severe combined immune deficiency) screening, performed during the last 2 years in an increasing number of countries, the USA in particular [6], [7]. Apart from great sensitivity in detecting SCID patients, this screening program repeatedly detected children with DiGeorge syndrome, as previously published [6], [7]. However, the population data suggest that only a minority of DiGeorge syndrome patients with the most severe T-lymphopenia can be identified via TREC screening. Analogous to T lymphocytes, newly developed B cells can be detected using kappa-deleting element recombination circles (KREC) [8]. A combined TREC/KREC approach has been able to identify patients with severe B cell disorders such as X-linked agammaglobulinemia in addition to SCID patients [9]. Various disorders in B cell function, supposedly originating from disturbed T and B cell interactions, have been described [1], [10]. In our study, we performed retrospective combined TREC/KREC monitoring with correction for control gene amplification in a cohort of patients with DiGeorge syndrome, and it enabled us to directly compare the results from neonatal dry blood spots with levels in peripheral blood postnatally. We aimed at finding out the percentage of DiGeorge syndrome patients who could be identified via TREC screening of newborns using our methodology. Furthermore, we tested whether addition of KREC could add more information both at birth and during the postnatal period in DiGeorge syndrome patients.

## Patients and Methods

### Patients

Thirty-two children (age 1.5–18 years) with DiGeorge syndrome with verified deletion of 22q11 and macroscopically missing/dysplastic/hypoplastic thymus were investigated. Del22q11.2 was verified either via FISH (fluorescent in-situ hybridisation) using the DiGeorge/VCFS TUPLE 1/22q Deletion Syndrome LPU004 probe (Cytocell, Cambridge, UK) or by using Salsa MLPA (Multiplex Ligation Probe Amplification) kits P-245-B1 or P-250-2B (MRC Holland). Twenty-eight of the 32 DGS patients underwent cardiac surgery. In 10 patients the thymus was reported as missing in the surgery protocol; in 4 patients the thymus was found to be hypoplastic and was subtotally resected; and in 3 patients only subtotal thymus resection was reported. In 10 patients, there was no information about thymic appearance; however, most of the remaining tissue was presumably also resected during surgery. Four patients had no surgery and were investigated and genetically verified based on their clinical symptoms. As controls, residual blood samples after routine clinical evaluations due to medical conditions that do not affect the lymphocyte count were used, or samples from healthy siblings of patients investigated at our department were used providing that they signed a written informed consent for the use of residual material (in total 91 children aged 1–18 years). Samples from five consecutively diagnosed patients with SCID in 2011–2013 in the Czech Republic (age of 2–4 months) were also evaluated. Archived neonatal DBSs were examined for 13 patients with DiGeorge syndrome and 10 patients with SCID. All patients or their parents provided written informed consent for the use of residual PB or DBSs in accordance with the Declaration of Helsinki. Fifty-six control DBSs were randomly chosen as controls, with an attempt to cover a DBS storage period similar to those of the patients. The birth weight of control newborns was recorded and subsequently the control DBSs were anonymised. The study was approved by the Ethics Committee of 2nd Medical School in Prague, Czech Republic.

### DNA isolation

DNA was isolated from 200 µl of peripheral blood using QIAamp DNA Blood Mini Kit (Qiagen GmbH, Hilden, Germany). From each DBS, a circle with a diameter of 3.2 µm was cut and DNA was eluted at 99°C for 1 hour in a shaker (500 rpm) using 100 µl of Generation DNA Elution Solution (Qiagen GmbH, Hilden, Germany) supplemented with 100 µg/ml of yeast tRNA (Life Technologies, Carlsbad, CA, USA).

### TREC and KREC detection

The albumin gene level was quantitatively detected in isolated samples using qPCR [11] and a standard dilution series derived from human genomic DNA with a known starting concentration (Roche, Basel, Switzerland). The levels of TREC and KREC signal joints were assessed separately using serial dilutions of cloned plasmid standards as previously described [8], [12]. One microlitre of DNA was added to each reaction. The results were subsequently recalculated to the levels of the albumin gene and expressed as the number of TREC (KREC) copies per one microgram of DNA.

### Statistical analysis

The distribution of variables between groups was assessed using the chi-squared or Fisher's exact test. The Mann-Whitney test was used to estimate the significance of the differences in continuous values. These statistical analyses were performed using StatView version 5.0 (StatView Software, Cary, NC). For evaluating TREC (KREC) dependence on age in patients and controls, a linear model was developed using R-package 3.1.1. (www.r-project.org).

## Results

### The levels of TREC/KREC in Guthrie cards

**Figure 1:**
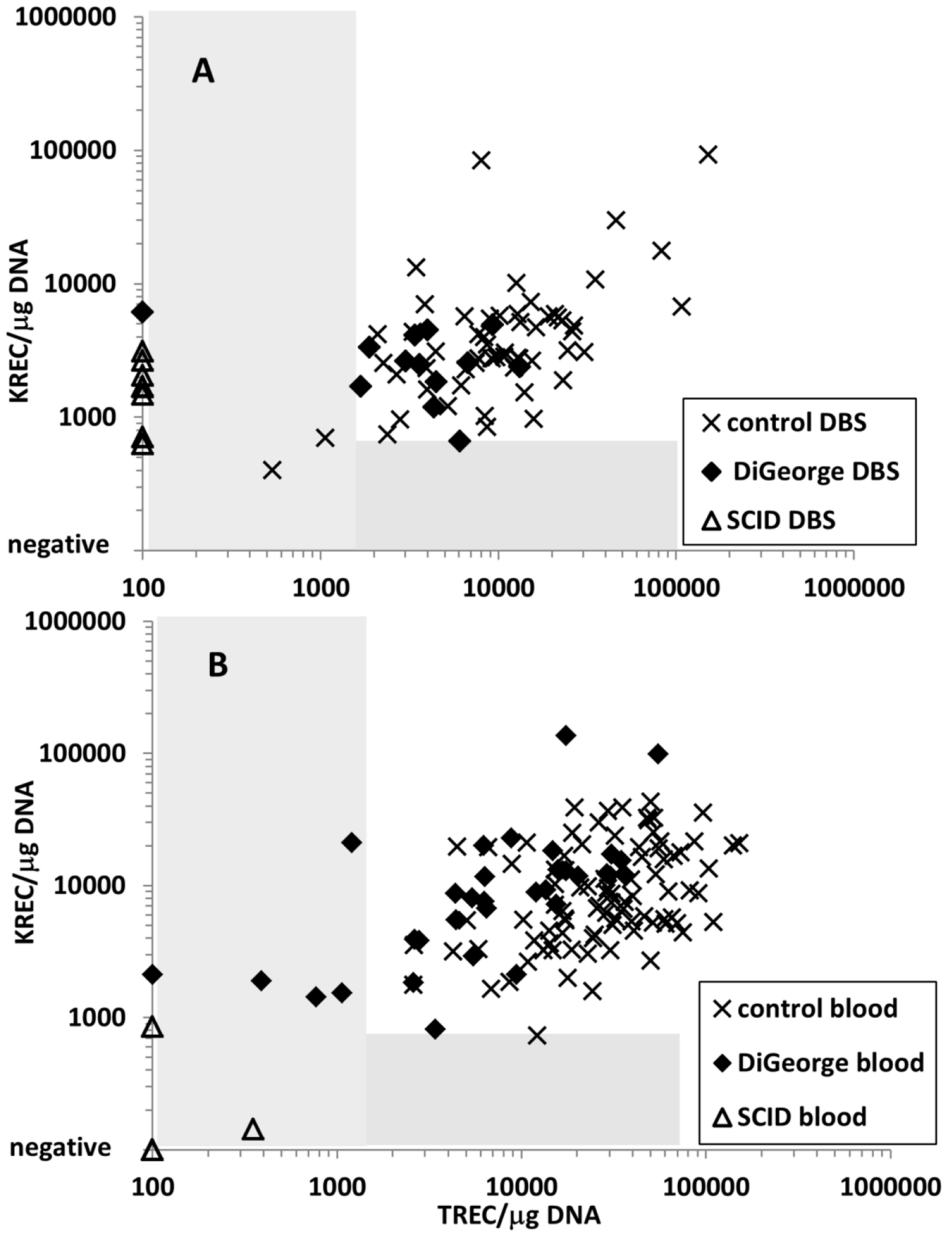
TREC and KREC numbers expressed as copies per microgram of DNA in A) neonatal dry blood spots B) peripheral blood in children (0–18 years) for controls, patients with DiGeorge syndrome and patients with severe combined immunodeficiency (SCID). Grey areas represent the abnormal TREC/KREC range.

The storage time of the DBSs was 1–18 years. The analysis of control gene amplification in DGS and SCID patients showed no difference between long- and short-term stored DBS (data not shown). First, we established ranges for normal and abnormal TREC and KREC levels. Most current approaches use either TREC alone or combined TREC/KREC detection without correction for DNA quality. Therefore, the numerical TREC/KREC values are not directly comparable between patients. We adopted an approach presented by Kamae et al [13]. Here, TREC/KREC copy numbers are expressed per one microgram of DNA, the DNA concentration being assessed by qPCR. We assessed TREC/KREC values in DBSs of 56 control newborns and in 10 patients with SCID. Three SCID patients were both TREC- and KREC-negative (2× RAG1 deficiency, 1× ADA deficiency). The remaining seven patients (6× common gamma chain mutation, 1× unknown mutation) had negative TRECs while their KREC levels were similar to controls (Figure 1A). Based on those findings, we set the cut-offs at 700 copies/µg DNA for KREC and 1400 copies/µg DNA for TREC. Of note, the control DBS with the lowest TREC/KREC values outside the determined range was from a premature child with birth weight of 890 grams. Figure S1 shows the same values copy numbers per one µl of dried blood without normalisation to a control gene.

**Figure 2:**
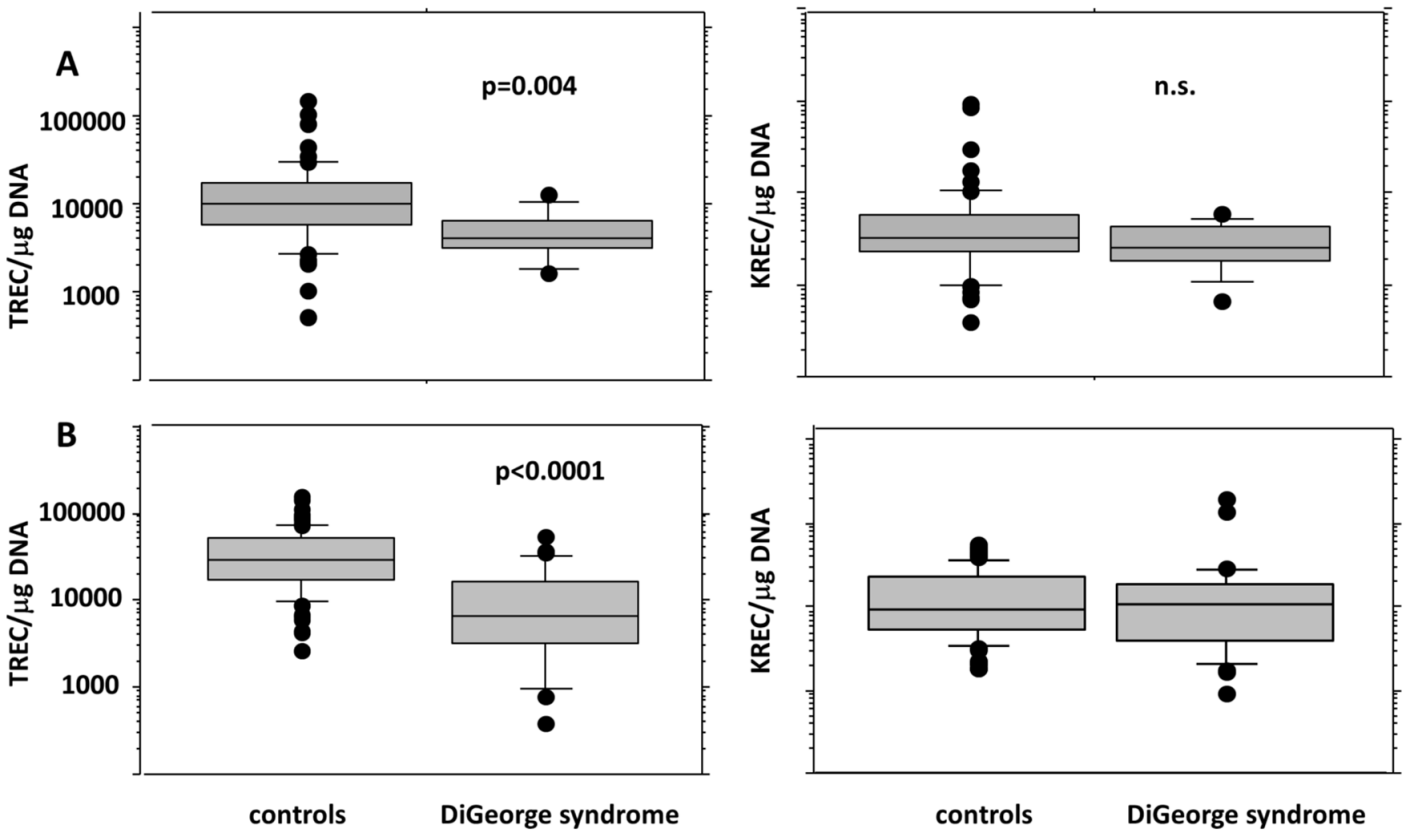
Quantitative TREC/KREC levels expressed as number of copies per microgram of DNA in A) neonatal dry blood spots B) peripheral blood for control children vs. children with DiGeorge syndrome (0–18 years).

Of the patients with DiGeorge syndrome, only one had completely negative TREC levels with normal KREC levels. This patient with complete athymia and the clinical picture of CHARGE syndrome received two DLIs from an unrelated donor at the age of six months [14]. Now, at the age of 9.5, the patient has reduced numbers of T lymphocytes with no naïve T cells, but suffers from no major infection complications. However, the TREC levels in his PB remain negative. The remaining twelve patients with DiGeorge syndrome had TREC levels within the normal range, and all but one also had normal KREC levels. One patient had KREC levels near the cut-off (664 copies/µg DNA). Quantitative TREC values were significantly lower in DiGeorge syndrome DBSs (Figure 2A), even after exclusion of the TREC-negative patient from the analysis (TREC: median 4181 in DiGeorge vs. 9924 copies/µg DNA in controls, p = 0.004, KREC: median 2552 in DiGeorge vs. 3156 in controls, n.s., Mann-Whitney).

### Postnatal TREC/KREC values and their development in children with DiGeorge syndrome

**Figure 3:**
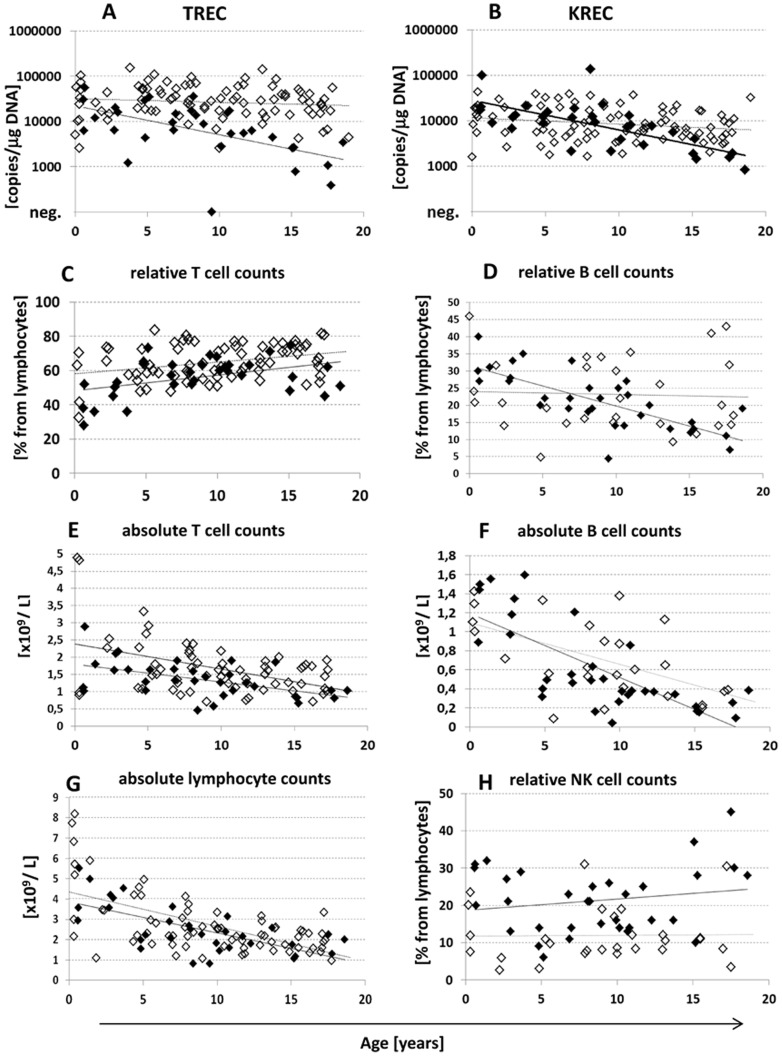
Quantitative levels of TRECs (A) KRECs (B), relative T cell (CD3^pos^) counts (C), relative B cell (CD19^pos^) counts (D), absolute T cell counts (E), absolute B cell counts (F), absolute lymphocyte counts (G) and relative NK cell (CD3^neg^CD16^pos^CD56^pos^) counts (H) in relation to age (x-axis) in patients with DiGeorge syndrome (black diamonds) and in controls (empty diamonds). Lines show linear regression analyses for patients (full lines) and controls (dotted lines).

The monitoring of TREC/KREC postnatally is used in research studies, but it is not routinely used in clinical examination of patients with immunodeficiency. We collected TREC/KREC values in a cohort of 91 control PB samples from children aged 0–18 years. Although there is a published trend of both TREC and KREC decrease postnatally [13], [15], [16], our data from healthy children show that KREC levels declined only slightly with age (test in linear model, p = 0.015, Figure 3B), whereas TREC levels remained practically stable until the age of 18 (p = 0.33, Figure 3A). More controls from different age groups would be necessary to precisely define the ranges for the respective KREC cut-offs. We therefore retained the KREC cut-offs for abnormal findings obtained from DBSs, but the declining trend has to be kept in mind. The absolute lymphocyte counts and absolute and relative B cell counts were not significantly different between DGS syndrome patients and controls (Figure 3G, F, D). As expected, the absolute and relative numbers of T lymphocytes were significantly lower in DGS patients than in controls (p = 0.03 and p = 0.0003, Mann-Whitney, Figure 3E, C).The percentages of NK cells were significantly higher in DGS syndrome patients (p<0.0001, Mann-Whitney, Figure 3H).

In the group of 32 children with DiGeorge syndrome aged 1.5 to 18 years, the TREC values were under the normal range in 5 of 32 (16%) patients (Figure 1B). Even after excluding the complete DiGeorge syndrome patient after DLI with continuously negative TREC levels, the quantitative TREC values remained significantly lower than in controls (median 6444 copies/µg DNA in DiGeorge syndrome vs. 29343 copies/µg DNA in controls, p<0.0001, Figure 2B). Interestingly, unlike in controls, the TREC levels declined significantly with age in DiGeorge syndrome patients (linear model, p<0.0001). The KREC levels were not significantly different from those of the controls (n.s., Figures 1B and 2B). However, the KREC decline during childhood was more pronounced in patients (test in linear model, p<0.0001) than in controls (p = 0.0047, Figure 3B).

The four patients with TREC values under the normal range had repeated respiratory infections in infancy. All of them later developed allergies and/or autoimmunity in a form of autoimmune thyroiditis or thrombocytopenia, autoimmune conditions typically associated with DiGeorge syndrome. Their status, however, is not markedly different from that of other patients with diGeorge syndrome.

In 12 patients, it was possible to monitor TREC/KREC development from birth to the present. Figure S2A shows that TREC levels remained stable (less than 10% difference, n = 4) or increased (1.4–9-fold, n = 6) in 10 of 12 (80%) patients. Similarly, the KREC levels were stable or increased in 75% of patients (Figure S2B). Thus, based on these data, no marked deterioration in postnatal lymphocyte output occurs in DiGeorge syndrome patients. Interestingly, the trend was consistent between TREC and KREC. The patients with a decrease in TREC had lower or stable KREC numbers, and vice versa.

### Feasibility of TREC/KREC analysis in postnatal peripheral blood for differential diagnostics of primary immunodeficiencies

Neonatal screening for SCID is not yet performed in the Czech Republic. During the period of this study, we investigated five patients with suspected SCID (age 2–4 months) based on clinical findings and flow cytometry results. The TREC/KREC analysis confirmed the SCID diagnosis in those patients (Figure 1B) within the time frame of one day, and preparations for transplantation of hematopoietic stem cells started while exploring the molecular genetic cause of the SCID.

## Discussion

In the retrospective part of our study we measured TRECs in newborn dry blood spots from patients with clinically diagnosed and genetically verified DiGeorge syndrome. However, we were only successful in using this method to detect DiGeorge syndrome in a patient with complete athymia and the clinical picture of CHARGE syndrome [14]. All the other patients with partial DiGeorge syndrome exhibited TREC levels within the normal range at newborn age. Of 993,724 infants examined in California newborn screening, only one complete and eight incomplete DiGeorge syndrome patients were identified. Given the incidence of the syndrome, the authors concluded that approximately 5% of the DiGeorge syndrome patients with the most severe T lymphopenia could be identified at birth via TREC screening [6]. Recently, a study similar to ours has been published using TREC/KREC detection without correction using a control gene [17]. The percentage of DiGeorge syndrome patients identified by neonatal screening was 19% in that study. We could only identify 8% of patients using our approach. This result may be skewed due to the low number of examined cases. Nevertheless, the data in both studies suggest that the more sensitive approach of the Swedish group also identified patients with less severe T-lymphopenia who did not require immune-restoring therapy despite suffering from severe and recurrent viral infections. This demonstrates the importance of cut-offs for reporting abnormal results, which should be widely discussed before the implementation of routine newborn screening programs.

Decreased T-lymphocyte counts during the critical period of the first few years of age in DiGeorge syndrome have been demonstrated in previously published studies [18]. We examined TRECs in patients from similar age groups. TRECs in DiGeorge syndrome have been previously investigated [15], [19], [20], and consistent with expectations they were found to be lower compared to controls. Another trend of gradual TREC decline with age was documented and published [15], which can possibly be explained by a gradual decrease in the importance of the thymus as a source of naïve T-lymphocytes as it is replaced by the division of T-lymphocytes [21]. However, our results are only partly consistent with the previously published data. In the control children, the TREC levels remained stable instead of declining during infancy (until 18 years), and we only observed a significant decrease of TREC levels with age in DGS patients. In our cohort we identified four patients with reduced TREC levels during childhood (13%), and they had repeated respiratory infections in infancy and later in life developed allergies and/or autoimmunity typical for DGS syndrome patients, but generally they did well without immune intervention and did not differ significantly from patients with normal TREC levels. Unfortunately, the Guthrie cards were not available for those patients. In the majority of DiGeorge syndrome patients, we observed no marked deviation from normal values either at birth or postnatally, even if the overall quantitative TREC values were significantly lower in both groups than in controls.

Our method uses correction for albumin gene levels for all the patients in the first-line test, which is different from most nationwide screening programs. This approach is more expensive (estimated costs ∼12 USD per sample) than the “TREC-only” approach with estimated cost of 4.22 USD per sample. Recently, a newborn SCID screening study was performed by a British group using the commercial TREC EnLite kit (Perkin Elmer), and it successfully detected all SCID cases in DBS [22]. The kit does not require the isolation of DNA, which is very convenient for nationwide screening due to ethical issues. In conclusion, our method is more suitable for research studies and postnatal monitoring than for nationwide screening, having the advantage of a more precise comparison between patients and controls and allowing the monitoring of TREC/KREC development postnatally. Comparing the levels between birth and the postnatal period, we observed a 1.4 to 9-fold increase in TREC levels in 50% of the patients. This is consistent with published data on CD3-positive cell development and with clinical observations in those patients, both showing improvements after the first years of life [18]. We can speculate that extrathymic tissue, namely tonsils as recently published [23], contributed to the generation of new T-lymphocytes in those patients because in most of the children the residual thymi were destroyed during the cardiac surgery.

KREC levels were normal in most patients at birth, which is consistent with a previous report [17]. Postnatal KREC levels in patients with DiGeorge syndrome have not been studied to date. An interesting observation of our study was quantitatively lower KREC levels in one patient at birth, which were found to be normalised at the age of 12 years (showing an 11-fold increase compared to birth). This is consistent with a previous study showing lower B cell numbers in the early years of life normalising later in some patients [24].

One of the major achievements of our study was establishing a robust platform for TREC/KREC testing not only in newborn screening but also for rapid postnatal differential diagnostics of severe immunodeficiency. One has to keep in mind that normalised TREC/KREC numbers in postnatal blood can be skewed in cases with clonal expansion of mature lymphocytes, e.g., in response to infections. Indeed, the TREC/KREC levels did not correlate well with relative T (B) lymphocyte counts (Figure S3A, B). We already showed the usefulness of normalised KREC monitoring as a surrogate marker in the monitoring of B cell reconstitution after stem cell transplantation [25], and we are convinced that despite its limitations, it is a very useful independent marker showing clearly whether B/T lymphopoiesis is retained. As the nation-wide neonatal screening of SCID is not planned in the Czech Republic at least for the next year, we continue to build a network of informed paediatricians and immunologists who should ensure that all infants with a suspect clinical picture and/or family history undergo rapid TREC/KREC screening.

## Conclusions

In our study, TREC levels only identified one severe case with deep lymphopaenia, which corresponds to 8% of the limited DiGeorge syndrome cohort. Most children with incomplete DiGeorge syndrome had TREC/KREC levels in the normal range both at birth and postnatally. Nevertheless, the decline of both TREC and KREC levels with age was more significant in DGS patients than in controls. Thus, the assay complements routine investigations and follow-up of DiGeorge patients and might reflect age-related immune changes based on the del22q11 background.

## Supporting Information

Figure S1 **TREC and KREC numbers expressed as copies per one µl of dry blood without control gene correction for controls, patients with DiGeorge syndrome and patients with severe combined immunodeficiency (SCID).**
(TIF)

Figure S2 **Changes in TREC (A) and KREC (B) values between birth and the present in children with DiGeorge syndrome (dotted lines).** The levels in control children are depicted by empty circles. Grey areas represent the abnormal TREC/KREC range.
(TIF)

Figure S3 **The correlation of TREC levels with relative numbers of T lymphocytes (A) and correlation of KREC levels with relative numbers of B lymphocytes (B) for DiGeorge syndrome patients (full diamonds) and controls (empty diamonds).**
(TIF)
