# Supplementary figures and images for: The TREC/KREC Assay for the Diagnosis and Monitoring of Patients with DiGeorge Syndrome
Source: PLoS One. 2014 Dec 8;9(12):e114514. doi: 10.1371/journal.pone.0114514 (PMC4259354; doi:10.1371/journal.pone.0114514)

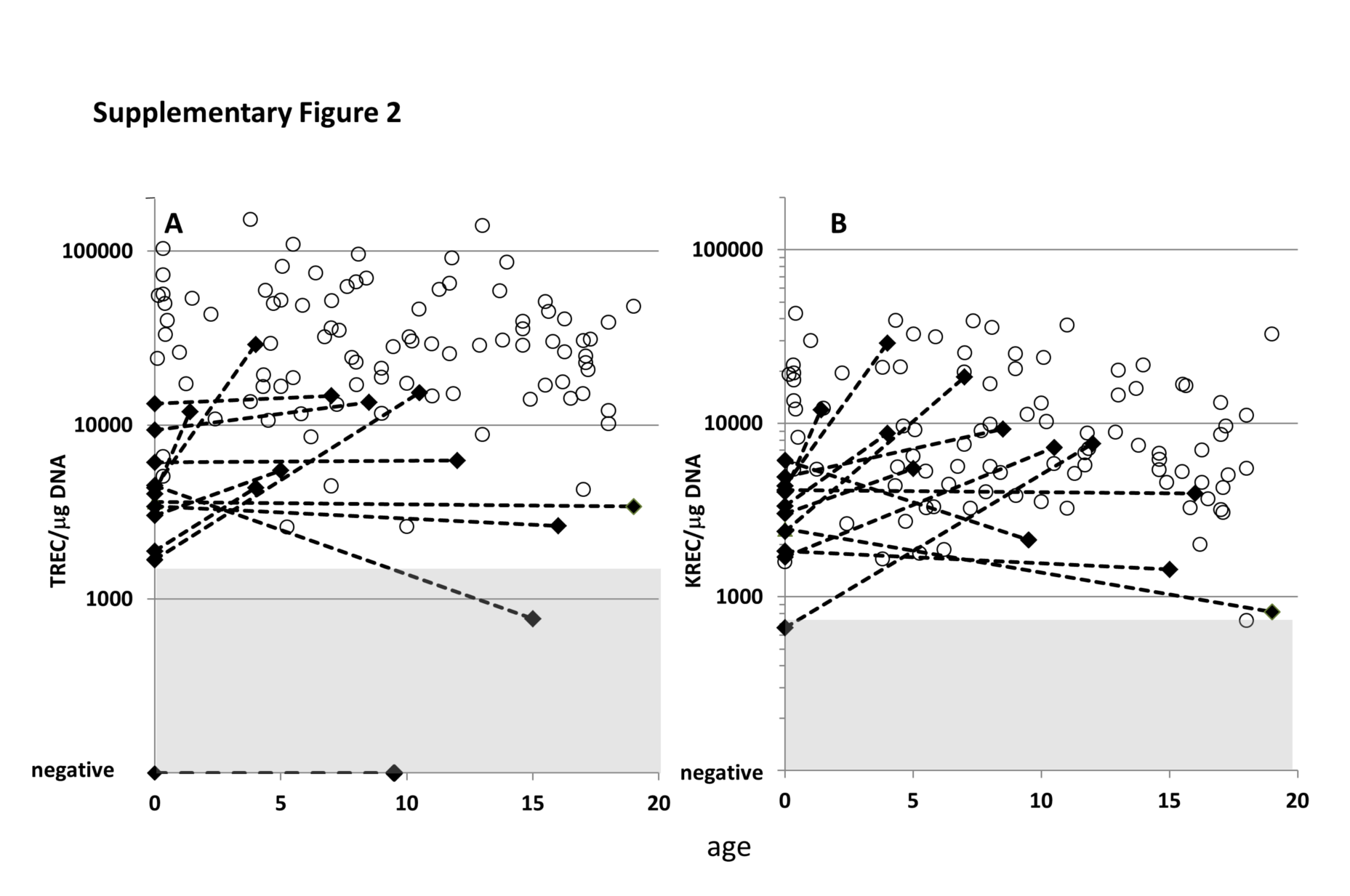

Supplement: Figure S2 — Changes in TREC (A) and KREC (B) values between birth and the present in children with DiGeorge syndrome (dotted lines). The levels in control children are depicted by empty circles. Grey areas represent the abnormal TREC/KREC range. (TIF) [file pone.0114514.s002.tif]

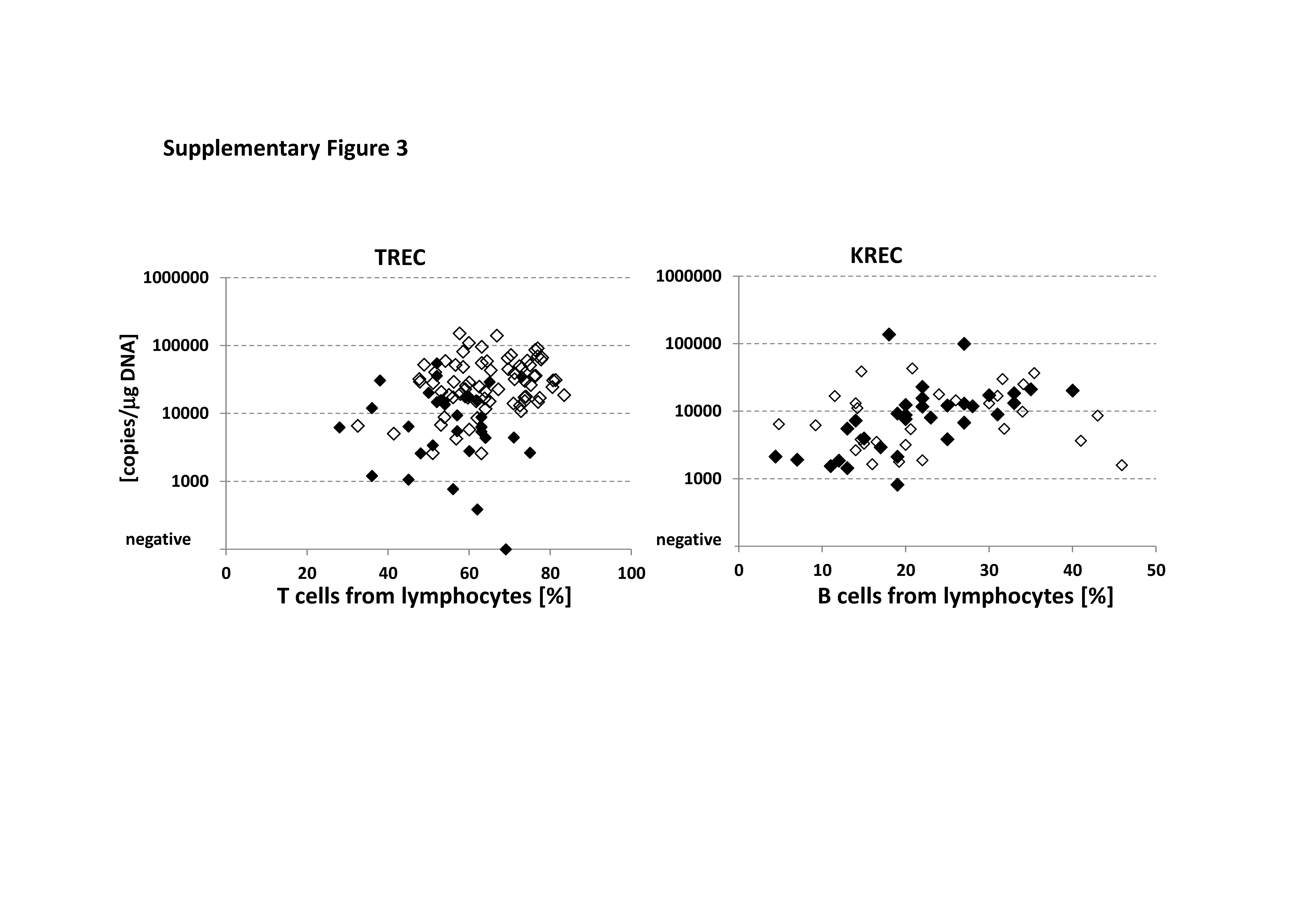

Supplement: Figure S3 — The correlation of TREC levels with relative numbers of T lymphocytes (A) and correlation of KREC levels with relative numbers of B lymphocytes (B) for DiGeorge syndrome patients (full diamonds) and controls (empty diamonds). (TIF) [file pone.0114514.s003.tif]
